# Pulsed Near-IR Photoresponse in a Bi-metal Contacted Graphene Photodetector

**DOI:** 10.1038/srep14803

**Published:** 2015-10-06

**Authors:** Xinghan Cai, Ryan J. Suess, H. Dennis Drew, Thomas E. Murphy, Jun Yan, Michael S. Fuhrer

**Affiliations:** 1 Center for Nanophysics and Advanced Materials, University of Maryland, College Park, MD 20742-4111 USA; 2 Institute for Research in Electronics and Applied Physics, University of Maryland, College Park, MD 20742 USA; 3 Department of Physics, University of Massachusetts, Amherst, MA 01003, USA; 4 School of Physics, Monash University, 3800 Victoria, Australia

## Abstract

We use an ultra-fast near-infrared pulse coincidence technique to study the time, temperature, and power dependence of the photoresponse of a bi-metal contacted graphene photodetector. We observe two components of the photovoltage signal. One component is gate-voltage dependent, linear in power at room temperature and sub-linear at low temperature-consistent with the hot-electron photothermoelectric effect due to absorption in the graphene. The power dependence is consistent with supercollision-dominated cooling in graphene. The other component is gate-voltage independent and linear in temperature and power, which we interpret as due to thermoelectricity of the metal electrodes due to differential light absorption.

The strong light-matter interaction in graphene^1^,^2^,^3^ makes it ideal for highly sensitive photodetection^4^,^5^,^6^,^7^,^8^. The excitation energy is transferred to an electrical signal output via the photovoltaic effect^9^,^10^, the photothermoelectric effect^11^,^12^,^13^ or the bolometric effect^14^,^15^,^16^. Photothermoelectric detectors are particularly promising for high speed, sensitive, broadband photodetection at room temperature^5^,^17^. In a graphene photothermoelectric detector, photo-excited charge carriers generate hot electrons due to electron-electron scattering^18^,^19^, and an asymmetry due to dissimilar metal contacts^5^,^20^, local gated p-n junctions^12^,^21^ or a bias voltage^22^ produces a net current via the thermoelectric Seebeck effect.

One critical process in the operation of a photothermoelectric device is the cooling of hot electrons, which limits both the detecting sensitivity and speed. Previous studies showed that in a graphene p-n junction photodetector, the hot electrons are mainly cooled by disorder-assisted phonon scattering processes termed supercollision^23^,^24^,^25^, whereas other studies concluded the direct emission of surface phonons of the polar substrate by graphene electrons plays an essential role^26^,^27^. While the nature of cooling in graphene p-n junction devices remains uncertain, there are no reports to date on the cooling processes in a technologically-relevant graphene-metal junction.

Here we study the cooling of hot electrons in a graphene based photodetector contacted with dissimilar metal electrodes (Cr and Au) that is uniformly illuminated by an ultrafast, pulsed near-IR excitation. We use the pulse coincidence measurement technique^28^ to study the time, power, and substrate temperature dependence of the photovoltage signal generated due to the hot-electron photothermoelectric effect with dissimilar metal contacts. Surprisingly, low-temperature pulse-coincidence measurements show either a peak (corresponding to an enhancement) or a dip (corresponding to an attenuation) in photoresponse when the pulses are coincident within the response time of the detector. The power dependent photoresponse measurement at low temperatures reveals that the photovoltage consists of a linear and a sub-linear component which may have different signs depending on the gate voltage, explaining the observation of both peaks and dips in the pulse-coincidence measurement. Further measurements at different temperatures show that the linear component is independent of the gate voltage, and is consistent with a thermoelectric effect in the contact metal, while the sub-linear component due to the absorption in graphene shows a power dependence consistent with the model based on supercollision cooling^23^,^24^.

The bi-metal contacted graphene photodetector is realized by exfoliating monolayer graphene on SiO_2_/Si substrate, followed by sequential Cr and Au metal electrode deposition using a standard e-beam lithography technique (see Methods). The inset of Fig. 1a shows the optical micrograph of the device; the graphene flake is contacted with Cr (Au) electrode on the left (right) side. Figure 1a shows the two-probe conductance *G* as a function of the applied back gate voltage *V*_g_ measured at *T* ~ 50 K. In the absence of an applied gate voltage, the device is *p*-doped and the charge neutral point is at *V*_g_ = 55 V. The two-probe field effect mobility of the device is ~500 cm^−2^∙V^−1^∙s^−1^, suggesting that the device is quite disordered. Next we characterized the photoresponse of the device at the same temperature using two near-IR (λ = 1.56 μm) pulsed laser beams (see Methods) with variable power and delay. The photovoltage at various gate voltages as a function of the delay time τ_d_ between the pump and probe pulses from −0.14 ns to 0.14 ns is plotted in Figs 1b,c. The feature at τ_d_ = 0 originates from the nonlinear nature of the photoresponse: If τ_d_ is much larger than the device’s intrinsic response time τ_r_, the device would have relaxed from the first excitation prior to the arrival of the second pulse, in which case the two pulses would generate two independent photoresponse signals to form the total photovoltage. When τ_d_ is comparable to or much smaller than τ_r_, their photoresponses cannot necessarily be linearly superposed, which enhances (weakens) the signal of the device when the response is super-linear (sub-linear) in power.

Figures 1b, c show the photoresponse as a function of delay time, for various gate voltages spanning the *p*-doped region (Fig. 1b) and *n*-doped region (Fig. 1c). Surprisingly, the two-pulse coincidence signal either shows a peak or a dip at zero delay time, depending on the applied gate voltages: for example in Fig. 1b, for *V*_g_ ≤ 25 V, the signal is enhanced when τ_d_ = 0, whereas for *V*_g_ ≥ 58 V, the signal decreases when two pulses temporally overlap each other. One possible explanation to this is that the signal is monotonically super-linear for *V*_g_ ≤ 25 V and sub-linear for *V*_g_ ≥ 58 V, resulting in an enhancement or an attenuation of the response at zero delay time, respectively. Another scenario that can account for the observed phenomenon is that the photoresponse consists of two components, one linear and the other nonlinear. The nonlinear signal contributes to the feature at τ_d_ = 0, while the linear part serves as an offset to the floor response, which if it has an opposite sign to the non-linear component, could change the polarity of the floor response, making the nonlinear enhancement/attenuation appear like an attenuation/enhancement.

To distinguish between these possibilities, the power dependence of the dc photoresponse was characterized at different temperatures using a single pulsed laser. Figure 2a shows the data taken at high temperature (*T* = 267 ± 2 K, where the error corresponds to fluctuations in temperature during the measurement of different data sets) and Fig. 2b plots the scaled photoresponse normalized by the incident power (which can be regarded as the responsivity in arbitrary units) as a function of the gate voltage. The fact, that at powers where the signal was well above the noise floor for all gate voltages, all curves coincide with one another in Fig. 2b suggests that the signal is proportional to the absorbed power (linear response) in this temperature range.

Figure 2c shows the power dependent photoresponse measured at low temperature (*T* = 120 ± 2 K). Compared to Fig. 2a, both the magnitude and the gate-voltage dependence of the signal have changed. More interestingly, it is found that the intersection point with the x-axis changes from *V*_g_ ~ 60 *V* to *V*_g_ ~ 70 *V*, as the incident light power gradually increases. This is shown in Fig. 2d, the zoomed-in plot of Fig. 2c. This indicates that at certain gate voltages the signal must be *non-monotonic* in power, in fact crossing zero at finite power. This evidence strongly suggests that the signal is composed of at least two components with different power dependences. The measured photovoltage, which is the summation of these two components, thus crosses the x-axis at different gate voltages when changing the incident power since both components have their own functional form of the gate voltage dependence.

To determine the origin of these two components of the signal, the temperature-dependent characterization of the photoresponse to one near-IR pulsed laser excitation is carried out and the results are shown in Fig. 3a. The temperature varied from *T* = 19.4 K to *T* = 201 K. The overall shape of the gate-voltage-dependent photovoltage changes only slightly with the temperature, while the major effect of temperature appears to be a uniform downward shift of the photovoltage along the y-axis with temperature. The simplest explanation for the observation is that the photovoltage is comprised of two components that separately depend on the gate-voltage and temperature, i.e., *V*_photo_(*V*_g_, *T, P*) = *V*_photo,1_(*V*_g_, *P*) + *V*_photo,2_(*T*, *P*). *T*o better understand the temperature dependence of *V*_photo_, the data shown in Fig. 3a is replotted as a function of the lattice temperature in its inset. It is easily seen that *V*_photo_ shows a linear dependence on the lattice temperature above 80 K. At low temperatures, the strong fluctuation^29^, which can also be observed in Fig. 3a, makes it difficult to discern the exact functional form of the signal vs. lattice temperature. Thus the data below 80 K is not shown.

Because the temperature-dependent component of the photovoltage barely changes when the carrier density of graphene is tuned over a wide range, we consider that this part of the signal is generated by light absorption occurring outside of the graphene flake. The reflectances, *R*, of chromium and gold at the wavelength λ = 1.55 μm are 0.66 and 0.98, respectively^30^. Considering that the transmission of the beam is very small for the thickness used in this device (~40 nm), the absorption in chromium pad is estimated to be as high as ~34%, which is much larger than graphene’s absorption (a few percent due to the interband transition) and the absorption in the gold pad. Therefore, it is possible that a thermoelectric response due to the absorption in chromium contributes to the total photovoltage signal of the device.

A control device of a chromium-gold thermocouple as shown in the inset of Fig. 3b is constructed to test this hypothesis (see Methods). The photothermoelectric response of the metal electrodes is characterized by focusing a CW near-IR (1.55 μm) laser beam on the device. The focused spot size is a few microns, so that local illumination is possible. The device is mounted in a cryostat and the photoresponse is measured at different temperatures as shown in Fig. 3b. The blue curve, which corresponds to the noise level, suggests that there is no photoresponse when the beam is focused on gold due to nearly 100% reflection of the surface. In contrast, a photoresponse, which shows a linear dependence of the temperature, is observed when the chromium surface is illuminated (red curve). This signal is further enhanced when the focused beam spot is adjusted closer to the Cr-Au junction. It is difficult to directly scale the photoresponse shown here to the temperature dependent component of the signal observed in the graphene photodetector shown in Fig. 3a, since both the laser source and the sample’s geometry have changed significantly. However, one can still make a qualitative estimation: The absorbed power of the chromium pad in Fig. 3b is comparable with the contact absorption in the experiment shown in Fig. 3a. However, the thermoelectric voltage is strongly reduced in Fig. 3b, because the wide Cr-Au junction (~700 μm in width) electrically shorts the light illuminated area (the spot size is ~3.5 μm), which behaves like a small battery, making the measured voltage ~200 (700 μm/3.5 μm) times smaller. This is not an issue for the data taken in Fig. 3a, because the spot size of the beam is large and covers the whole area of the bowtie electrodes. It is thus reasonable that the photovoltage shown in Fig. 3a is two orders of magnitude larger than that shown in Fig. 3b. A quantitative comparison requires considering more factors, such as the various heat pathways^31^,^32^ for both geometries and the difference between pulsed and CW excitations^24^. Nonetheless, the fact that chromium can absorb near-IR light and generate a thermoelectric response that is linear with the temperature suggests that the temperature-dependent component of the signal observed in the graphene detector is likely generated due to the chromium contact’s absorption.

Lastly, we consider the power dependence of *V*_photo_. According to the analysis in previous paragraphs, the photovoltage results from two components. The first, *V*_photo,1_ results from the thermoelectric effect in the electrodes, and should be linear in temperature and power. The second, *V*_photo,2_, is assumed to have a power-law power dependence, and depend on gate voltage:


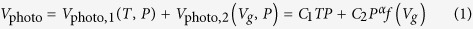


We subtract the signal at *V*_g_ = 35 V, where we observed a flat response in the pulse coincidence measurement, from each curve shown in Fig. 2c to obtain only the nonlinear component of the response *V*_photo,2_, while the subtracted gate-independent value is the linear component *V*_photo,1_.

Figure 4a shows the power dependence of the subtracted component *V*_photo,1_, which is indeed linear in power, consistent with the thermoelectric effect in the electrodes. Figure 4b shows the result of a power-law fit to the power dependence of the remaining *V*_photo,2_ at each gate voltage: the power law exponent α < 1 indicating a sublinear power dependence. The exponent α varies within a range from 0.65 to 0.95, consistent with the supercollision model in graphene, which predicts^24^ α varying from 0.5 to 1, depending on the energy per laser pulse. Note that this analysis is not performed between *V*_g_ = 35 V ~ 55 V due to the small signal (see below) which produced large errors in the fitting.

Figure 4c shows the gate-voltage dependence of *V*_photo,2_ at various powers. We see that *V*_photo,2_ changes sign twice with gate voltage, at approximately *V*_g_ = 35 and 55 V. We recall that *V*_photo,1_ is independent of gate voltage, hence the relative sign of *V*_photo,1_ and *V*_photo,2_ is the same for 35 V < *V*_g_ < 55 V, and opposite for *V*_g_ < 35 V and *V*_g_ > 55 V. Figure 4d replots the pulse-coincidence data from Figs 1b,c for comparison with Fig. 4c. For 35 V < *V*_g_ < 55 V the pulse-coincidence signal displays a dip feature at zero delay time, indicating a sub-linear power dependence. This is consistent with the signal being the sum of *V*_photo,1_ (linear) and *V*_photo,2_ (sub-linear) of the same sign in agreement with Fig. 4c. For *V*_g_ < 35 V and *V*_g_ > 55 V, the pulse-coincidence signal displays a peak feature at zero delay time; this is in agreement with the signal corresponding to the sum of a linear *V*_photo,1_ and sub-linear *V*_photo,2_ of *opposite sign*, resulting in a *super-linear* power dependence at high power. Again, this region corresponds well with the observation of a negative *V*_photo,2_ in Fig. 4c.

## Discussion

The graphene-metal junction is a complicated optoelectronic system, with each part of the device interacting with the incident power and contributing to the electric output of the circuit. In this work, we analyzed the photoresponse of a dissimilar metal contacted graphene photodetector as a function of gate voltage, temperature, and power, using near-IR pulsed radiation. We were able to successfully decouple the two components of the signal, one generated by graphene’s absorption and the other due to the absorption in the contact, by taking advantage of their different power, temperature and gate dependences. Specifically, we find that absorption by the electrodes results in a photovoltage that is linear in temperature and power, and independent of gate voltage. Absorption by the graphene, in contrast, results in a photovoltage with complex gate-voltage dependence, and a sub-linear power dependence consistent with supercollision cooling of hot carriers in the graphene.

Our simple decoupling method has captured the main operation principle of the device. However, some detailed questions still remain for discussion. For example, the heated chromium pad can generate a temperature gradient in graphene from Cr side to Au side, which contributes to a response that is linear in power (since Δ*T* is linear in power due to chromium’s absorption) but gate-dependent (due to the gate-dependent thermoelectric effect in graphene). This signal is generated in graphene, but due to the absorption in chromium, which is not considered in our simple model. This probably accounts for the observation that α depends on the gate voltage (Fig. 4b), not expected in the simple model.

Furthermore, some of previous work suggests that the photovoltaic effect also contributes to the photovoltage signal^9^,^33^, when the excitation photon energy is high enough to generate electron-hole pairs in graphene. In contrast to the gate-voltage-independent thermoelectric response of the contacts, the expected graphene photovoltaic signal should be gate dependent. A previous study^22^ in a biased graphene photodetector shows that the photovoltaic signal plays an essential role near the charge neutral point, while it drops off quickly as the carrier density of the graphene increases. This opens the possibility that *V*_photo,2_ is due, in part, to a photovoltaic effect in graphene. However, in this work, the gate dependence of the decoupled nonlinear component of the signal is consistent at all measured temperatures, with the simplest explanation that the signal is purely thermoelectric in origin, rather than consisting of two parts (thermoelectric and photovoltaic). Since there is a lack of reports on the power dependence of the photovoltaic response at different temperatures^22^,^34^,^35^, further studies will be needed to quantitatively determine the magnitude of the signal according to the different processes.

## Methods

**Figure 1:**
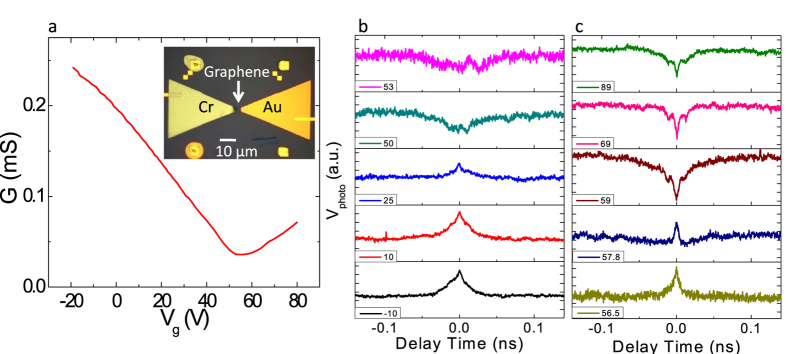
Transport and the pulse-coincidence measurement of a bi-metal contacted graphene photodetector. (**a**) Two-probe conductance as a function of the gate voltage. Inset: The optical micrograph of the device. (**b**,**c**) Photoresponse measured using the pulse-coincidence technique as a function of the delay time at p-doped (**b**) and n-doped (**c**) regions; legends indicate the gate voltage in volts. The temperature is fixed at *T* = 50 K. The unit scale of the y-axis is the same for all curves.

**Figure 3:**
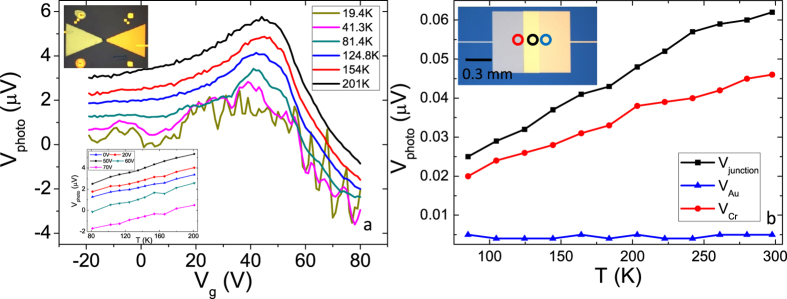
Temperature dependent photoresponse of the photodetector and a thermocouple. (**a**) Photovoltage as a function of the gate voltage to one pulsed near-IR laser excitation at different temperatures. Upper inset: Device’s optical micrograph replotted from the inset of Fig. 1a. Bottom inset: Photovoltage as a function of the temperature at different gate voltages. (**b**) Photoresponse of a Cr—Au thermocouple to a CW near-IR excitation as a function of the temperature. Data is shown for the beam focused on the gold pad (blue line), the chromium pad (red line), and the junction (black line). Inset: Optical micrograph of the thermocouple. Colored circles show the beam positions for curves in (**b**), correspondingly.

**Figure 4:**
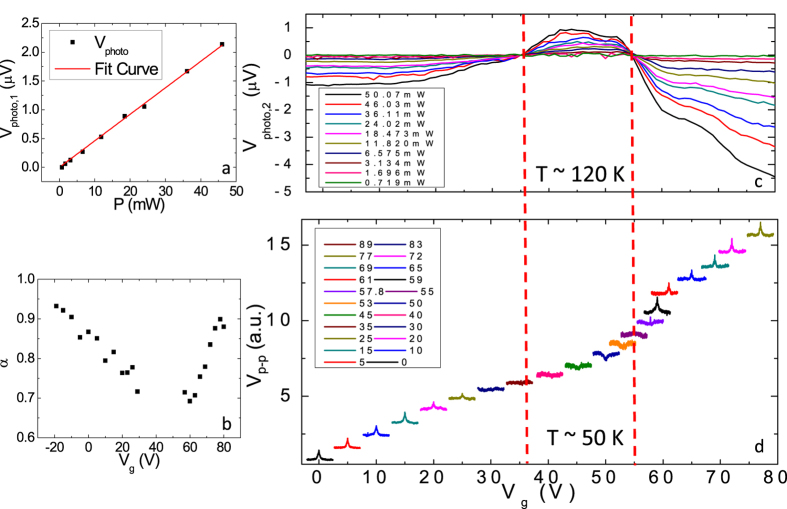
Decoupled photoresponse. (**a**) Linear component of the photovoltage as a function of the incident laser power (black dots) with a linear fit (red line). (**b**) Extrapolated α at different gate voltages. (**c**) Extrapolated nonlinear component of the photovoltage as a function of the gate voltage due to a pulsed near-IR laser excitation with different incident powers. (**d**) Photoresponse measured using the pulse-coincidence technique as a function of the delay time at different gate voltages; legends indicate the gate voltage in volts. *T* ~ 120 K for (**a**–**c**) and *T* ~ 50 K for (**d**).

Single-layer graphene was exfoliated from bulk graphite onto a substrate of 300 nm SiO_2_ over ion-implanted intrinsic Si. Chromium/gold electrodes (thickness 4 nm/45 nm), the chromium bowtie contact (thickness 35 nm), and the gold bowtie contact (thickness 40 nm) are thermally evaporated for the device shown in Fig. 1 in three lithographic steps. The liftoff mask is patterned via e-beam lithography using a bilayer resist [methyl methacrylate (8.5%)/methacrylic acid copolymer (MMA), Micro Chem Corp.; and poly(methy methacrylate) (PMMA), Micro Chem Corp.]. The chromium/gold thermocouple shown in Fig. 3b is fabricated using the same lithography technique as described above.

The device is mounted in a continuous flow cryostat system (*Janis Research*) to characterize the temperature dependence of the photoresponse from room temperature down to ~10 K. The response of the graphene photodetector shown in Fig. 1a is characterized by *Menlo Systems C-Fiber Fiber Laser*, which outputs 1.56 μm pulsed excitations with a pulse width of ~60 fs at a repetition rate 100 MHz. The average power of the beam can be tuned up to ~50 mW. The photoresponse is characterized by illuminating the detector with a chopped laser beam and detecting the open-circuit photovoltage signal using a voltage preamplifier and lock-in amplifier. The beam is focused on the detector using a glass lens with the beam size a few hundred microns in diameter. The photoresponse of the thermocouple shown in Fig. 3b is characterized in a similar way. The only difference is that the excitation source is *81663A Distributed Feedback Laser (Keysight Technologies)*, which is a CW near-IR laser with wavelength 1.55 μm and a maximum output power ~20 mW, and the beam is focused to a few microns in diameter.

The pulse coincidence measurement is characterized by two *Menlo Systems C-Fiber Lasers*. The repetition rate of the pump pulse is *f*_*0*_ = 100 MHz, which is slightly different from the probe pulse with a repetition rate *f* = *f*_*0*_ + δ*f*, resulting in an asynchronous illumination on the device. The range of the delay time varies from −0.14 ns to 0.14 ns.

## Additional Information

**How to cite this article**: Cai, X. *et al.* Pulsed Near-IR Photoresponse in a Bi-metal Contacted Graphene Photodetector. *Sci. Rep.* **5**, 14803; doi: 10.1038/srep14803 (2015).

## Figures and Tables

**Figure 2:**
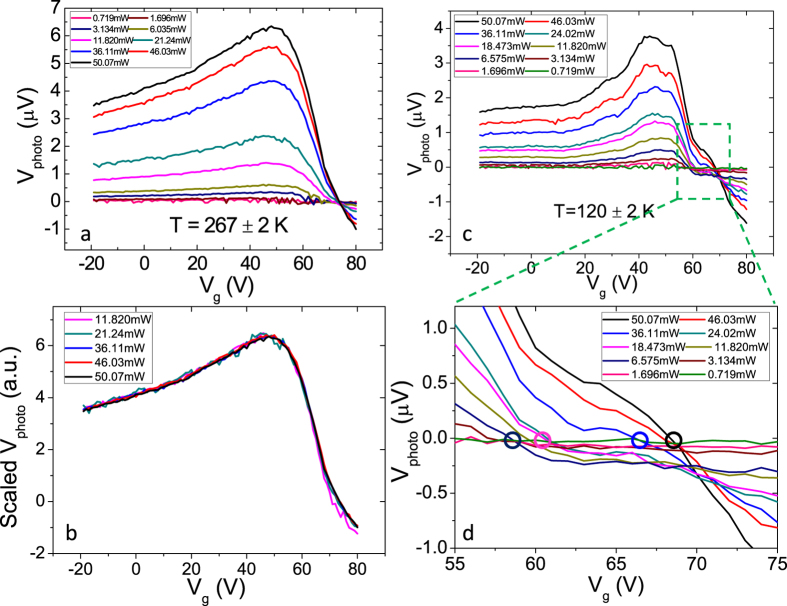
Power dependence of the photoresponse. (**a**,**c**) Photovoltage as a function of the gate voltage due to one pulsed near-IR laser with different average incident powers as shown in legend. The temperatures are *T* = 267 ± 2 K (**a**) and *T* = 120 ± 2 K (**c**). (**b**) Photoresponse shown in (**a**) normalized by the incident power as a function of the gate voltage. (**d**) Zoomed-in plot of (**c**) showing the response from *V*_g_ = 55 V to *V*_g_ = 75 V. Zero crossings of several selected curves are marked with corresponding colored circles.
